# Plumbagin-Loaded Nanoemulsion Drug Delivery Formulation and Evaluation of Antiproliferative Effect on Prostate Cancer Cells

**DOI:** 10.1155/2018/9035452

**Published:** 2018-11-11

**Authors:** Adrian Chrastina, Veronique T. Baron, Parisa Abedinpour, Gaelle Rondeau, John Welsh, Per Borgström

**Affiliations:** Vaccine Research Institute of San Diego (VRISD), San Diego Science Center, San Diego, California, USA

## Abstract

**Background:**

Plumbagin, a medicinal plant-derived 5-hydroxy-2-methyl-1,4-naphthoquinone, is an emerging drug with a variety of pharmacological effects, including potent anticancer activity. We have previously shown that plumbagin improves the efficacy of androgen deprivation therapy (ADT) in prostate cancer and it is now being evaluated in phase I clinical trial. However, the development of formulation of plumbagin as a compound with sparing solubility in water is challenging.

**Methods:**

We have formulated plumbagin-loaded nanoemulsion using pneumatically controlled high pressure homogenization of oleic acid dispersions with polyoxyethylene (20) sorbitan monooleate as surfactant. Nanoemulsion formulations were characterized for particle size distribution by dynamic light scattering (DLS). The kinetics of* in vitro* drug release was determined by equilibrium dialysis. Anticancer activity toward prostate cancer cells PTEN-P2 was assessed by MTS (Owen's reagent) assay.

**Results:**

Particle size distribution of nanoemulsions is tunable and depends on the surfactant concentration. Nanoemulsion formulations of plumbagin with 1-3.5% (w/w) of surfactant showed robust stability of size distribution over time. Plumbagin-loaded nanoemulsion with average hydrodynamic diameter of 135 nm showed exponential release of plumbagin with a half-life of 6.1 h in simulated gastric fluid, 7.0 h in simulated intestinal fluid, and displayed enhanced antiproliferative effect toward prostate cancer cells PTEN-P2 compared to free plumbagin.

**Conclusion:**

High drug-loading capacity, retention of nanoparticle size, kinetics of release under simulated physiological conditions, and increased antiproliferative activity indicate that oleic-acid based nanoemulsion formulation is a suitable delivery system of plumbagin.

## 1. Introduction

**Figure 1:**
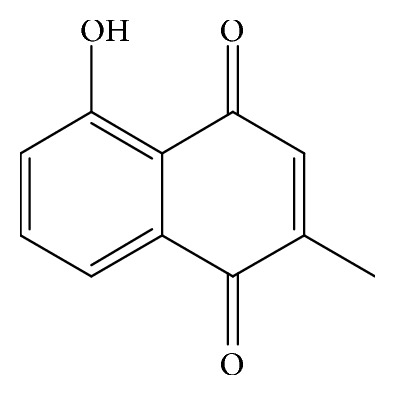
Chemical structure of plumbagin (5-hydroxy-2-methyl-1,4-naphthoquinone or 5-hydroxy-2-methyl-1,4-naphthalenedione, C_11_H_8_O_3_).

Plumbagin (5-hydroxy-2-methyl-1,4-naphthoquinone, Figure 1) is a plant-derived secondary metabolite found in plant families such as Plumbaginaceae, Ebenaceae, Dioncophyllaceae, Ancestrocladaceae, and Droseraceae [1]. Plumbagin possesses multiple pharmacological activities, such as anticancer, antiatherosclerotic, antidiabetic, anti-inflammatory, antimicrobial, hypolipidemic, and neuroprotective activities [2, 3]. Recently, significant research effort was developed to evaluate antitumor effects of plumbagin. Plumbagin showed potent anti-tumor activity in various tumor models, including breast cancer [4], Ehrlich ascites carcinoma [5], esophageal cancer [6], lung cancer [7], melanoma [8], ovarian cancer [9], promyelocytic leukemia [10], and prostate cancer [11–13]. Plumbagin exhibits these anticancer effects via interaction with multiple targets and modulation of various molecular signaling pathways, including AMPK, CDK1/CDC2, cyclin B1, cyclin D1, NF-kappaB, p53, p21 Waf1/Cip1, p27 Kip1, Nrf2/ARE, PI3K/AKT/mTOR, Ras, STAT3/PLK1/AKT, and Wnt [3], resulting in induction of apoptosis and autophagy, cell cycle arrest, inhibition of invasion, and metastasis, as well as antiangiogenic activities [2].

Great progress has recently been made regarding its use in prostate cancer. For example, experiments from Dr. Verma's laboratory demonstrated that plumbagin administered by intraperitoneal injection strongly inhibited tumor growth or delayed disease progression in several models of prostate cancer including subcutaneous xenografts, hormone-independent orthotopic tumors, transgenic TRAMP/FVB, and knock-out PTEN^−/−^ mouse models [14–16]. It also decreased the occurrence of metastases and therefore may be effective for the treatment of metastatic castration-resistant prostate cancer [15]. In addition, plumbagin considerably improved the efficacy of castration and chemical ADT in hormone-sensitive models of prostate cancer, causing tumor regression and increasing mouse survival when used in combination [11, 12]. IND-enabling preclinical studies were completed, leading to the first-in-human clinical trial for the combination of plumbagin and ADT in prostate cancer patients (NCT03137758).

Translation of plumbagin into clinical application unfortunately is made difficult by its poor water solubility. Drug solubility fundamentally influences its pharmacokinetics, its rate and extent of absorption, and its bioavailability after peroral administration [17], and thus essentially its therapeutic efficacy. As the drug advances into the clinic, it becomes critical to develop a formulation that considerably increases its solubility while potentially reducing systemic toxicity. Indeed, a better formulation would improve the patient's quality of life and achieve optimal therapeutic potential. Here, we describe the development and characterization of a nanoemulsion formulation of plumbagin. Nanoemulsions are kinetically stable, optically isotropic, biphasic dispersions of oil-in-water stabilized by an interfacial film of amphiphilic surfactant [18]. These nanoparticle systems have droplets within submicron size range, typically 5-200 nm. Nanoemulsions have multiple potential advantages for oral delivery of active pharmaceutical ingredients, including high encapsulation capacity for poorly water soluble drugs, robust stability to separation, flocculation and coalescence, and potentially improved absorption and bioavailability of encapsulated drugs [19, 20]. We have formulated nanoemulsions using biocompatible components, with oleic acid as an oil phase and polyoxyethylene (20) sorbitan monooleate (polysorbate 80) as an emulsifier. High plumbagin-loading capacity, kinetics of release in simulated physiological environments, and enhancement of antiproliferative activity toward prostate cancer cells suggest that oleic-acid based nanoemulsion formulation of plumbagin is a suitable delivery system.

## 2. Materials and Methods

### 2.1. Materials

Plumbagin, polysorbate 80, oleic acid and all other chemicals were supplied by Sigma (St. Louis, MO, USA) unless otherwise stated.

### 2.2. Preparation of Plumbagin-Loaded Nanoemulsion

To find the optimal composition for a formulation of plumbagin-loaded nanoemulsions, series of samples with increasing concentrations of surfactant were processed by high-pressure homogenization. Briefly, coarse emulsions of oleic acid (10% w/w) saturated with plumbagin, prepared in 0.25-4.0% (w/w) polysorbate 80 in deionized water, were homogenized using high speed homogenizer (Biospec Products, Inc.) at 30,000 rpm for 5 min. Emulsions were then processed by high-pressure homogenization using pneumatically controlled EmulsiFlex-C3 (Avestin) 10 cycles at 5000 psi.

### 2.3. Particle Size Analysis

Nanoemulsion formulations at the time of production and at different time points afterwards were analyzed for particle size distribution and polydispersity by dynamic light scattering (DLS) method using noninvasive back scatter (NIBS) detection at 173° angle and at 25°C on Zetasizer Nano-ZS (Malvern Instruments) equipped with 4 mW He-Ne, 633 nm laser.

### 2.4. Physical Stability of Nanoemulsions

Plumbagin-loaded nanoemulsions were evaluated for any particle size change over time, or after incubation in 0.1M HCl or 0.01M sodium phosphate buffer pH 6.8 or pH 7.5, simulating physiological conditions. Particle size distribution was analyzed at designated time-points using Zetasizer Nano-ZS (Malvern Instruments) as described above.

### 2.5. In Vitro Drug Release

*In vitro* plumbagin release from nanoemulsion formulation was performed using membrane dialysis (MW cutoff 8-10 kDa) against simulated gastric fluid (0.1M HCl, 0.05% (w/v) Tween 80) or simulated intestinal fluid (0.01M sodium phosphate buffer pH 7.5, 0.05% (w/v) Tween 80) at 37°C. At designed time intervals, the concentration of plumbagin was determined by UV-VIS spectrometry from four independent measurements (n=4) as described below. To analyze kinetics of release, the data were fitted by one phase exponential nonlinear regression using GraphPad PRISM 6.

### 2.6. Determination of Plumbagin Concentration

Concentration of plumbagin in nanoemulsions was estimated by UV-VIS spectrophotometry. Briefly, nanoemulsions were dispersed in methanol (200-3000 X dilution) vortexed for 10 sec and then concentration of plumbagin was determined spectrophotometrically (DU-640, Beckman Coulter) at 410 nm (*ε*_*λ*_ = 3,800 dm^3^.mol^−1^.cm^−1^).

### 2.7. Cell Culture

PTEN-P2 murine prostate cancer cells were kindly provided by the Wu laboratory and have been previously described [21]. The cells were grown in RPMI-1640 medium (Sigma) supplemented with 10% (v/v) heat-inactivated fetal bovine serum, 2 mM L-glutamine, 100 U/ml penicillin, 100 *μ*g/ml streptomycin, insulin-selenium-transferrin (5 *μ*g/ml insulin), and 10^−8^ M dihydrotestosterone. Cultures were passaged by dissociation using trypsin (0.05%) and maintained at 37°C in a humidified atmosphere with 5% CO_2_.

### 2.8. In Vitro Cytotoxicity Assay

The antiproliferative properties of plumbagin nanoemulsion were examined by MTS tetrazolium compound (Owen's reagent) based assay. Briefly, the PTEN-P2 cells were plated at density of 8 × 10^3^ cells per well of 96-well plates in four replicates. 24 h later, the medium was replaced with medium containing increasing concentration of plumbagin or nanoemulsion formulation of plumbagin corresponding to the range of 0-10 uM plumbagin. Control nanoemulsion formulation without drug was tested at the same concentration range but without plumbagin. After 24 h exposure, the cytotoxicity was determined using a CellTiter 96AQueous on solution cell proliferation assay (MTS) kit according to manufacturer's instructions (Promega, Madison, WI) by measuring conversion of (3-(4,5-dimethylthiazol-2-yl)-5-(3-carboxymethoxyphenyl)-2-(4-sulfophenyl)-2*H*-tetrazolium, inner salt) to formazan product. IC_50_ values were then determined from cytotoxicity curves as a concentration that induced 50% inhibition of the cell growth.

### 2.9. Data Analysis

Particle size (Z-average, intensity-weighted mean size) and polydispersity index (PDI) were determined by method of cumulants (defined by ISO standards 13321 and 22412) of autocorrelation function generated by DLS instrument. Drug release and cytotoxicity studies were performed in replicates (n=4) and the %cumulative release and %cytotoxicity values are expressed as means from these measurements with corresponding standard deviations.

## 3. Results

### 3.1. Formulation of Plumbagin-Loaded Nanoemulsions

**Figure 2:**
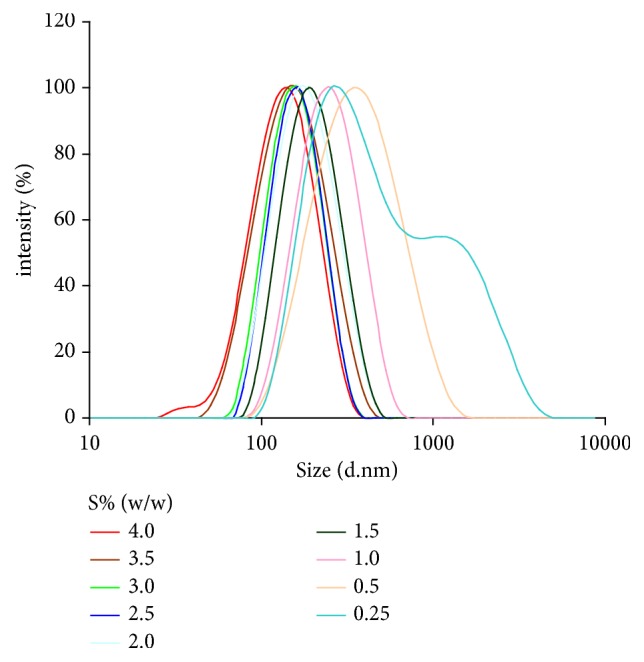
Particle size distribution profile of plumbagin-loaded nanoemulsions is tunable and depends on surfactant concentration (S% (w/w)) during processing of emulsion by high pressure homogenization. Particle size is indicated as intensity-based Z-average hydrodynamic diameter (nm) determined from DLS measurements.

**Figure 3:**
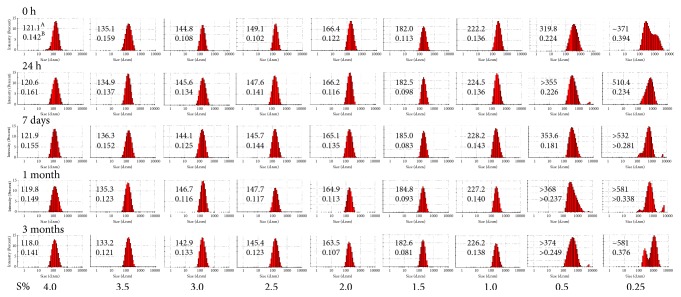
Time-dependent intensity-based particle size distribution profiles of plumbagin-loaded microemulsions at different surfactant concentrations (S%, w/w). A: particle size is indicated as Z-average hydrodynamic diameter (nm) determined by cumulants fit analysis from DLS measurements. B: heterogeneity of size distribution is indicated by polydispersity index (PDI).

Our preformulatory tests of various excipients identified oleic acid to be a good solubilizer of plumbagin (51.8 mg/ml at 25°C). Therefore, we proceeded to optimize the formulation of plumbagin nanoemulsions using oleic acid as the oil phase. Dispersions of oleic acid saturated with plumbagin were generated by high-pressure homogenization of coarse emulsions with variable contents of polysorbate 80 as surfactant. Particle size analysis by DLS showed a decrease of hydrodynamic diameter with increasing concentration of surfactant, as indicated by intensity-based particle size distribution profiles (Figure 2). Dispersions with concentrations of polysorbate 80 equal to or less than 0.5% (w/w) showed high polydispersity index as also indicated by the wide range of particle size distribution (Figure 3). Furthermore, these dispersions were unstable over time, showing multimodal distribution patterns with presence of particles with a diameter higher than 1000 nm (Figure 3). Progressive increase in surfactant concentration above 1.0% (w/w) stabilized dispersions. Nanoemulsions with surfactant content in the range of 1.0-3.5% (w/w) showed narrow particle size distribution profiles and high stability over time (Figure 3). Dispersions with higher concentrations of polysorbate 80 (4.0% w/w) showed the smallest average radius, although we observed the presence of ~ 30 nm surfactant micelles in these formulations. Therefore, a surfactant content in the range of 1.0-3.5% yielded uniform plumbagin-loaded nanoemulsions with an average particle size that can be controlled by the concentration of surfactant during high-pressure homogenization.

### 3.2. Stability of Nanoemulsions

**Figure 4:**
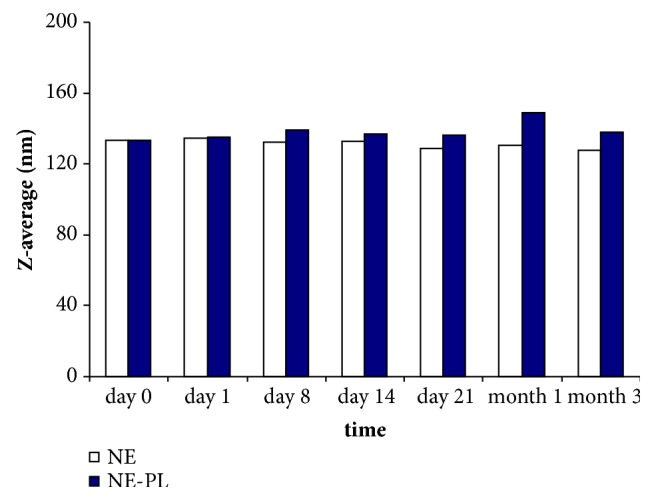
Stability of size distribution profile of oleic acid-based nanoemulsions (polysorbate 80 (3.5%) without plumbagin (NE) and plumbagin-loaded (NE-PL) nanoemulsion formulation at 25°C over time.

Nanoemulsions with narrow polydispersity and diameter (Z-average, 135 nm, 3.5% (w/w) polysorbate 80) were selected for further characterization. This formulation of plumbagin-loaded nanoemulsions and the same formulation without plumbagin showed nearly identical particle size immediately after preparation (Figure 4). Both plumbagin-loaded and control (empty) nanoemulsions showed good retention of size distribution over three months of storage at 25°C (Figure 4), with only a moderate increase of diameter of the plumbagin-loaded nanoemulsions. Furthermore, both the control nanoemulsion and the plumbagin-loaded nanoemulsion, when dispersed in acidic media that simulate the physiological environment of stomach acid and intestinal fluids, showed retention of particle size (Table 1). A moderate increase in the hydrodynamic diameter of both empty- and plumbagin-loaded nanoemulsions was observed in the slightly alkaline environment of phosphate buffer (pH 7.5) (Table 1).

### 3.3. In Vitro Release of Plumbagin

**Figure 5:**
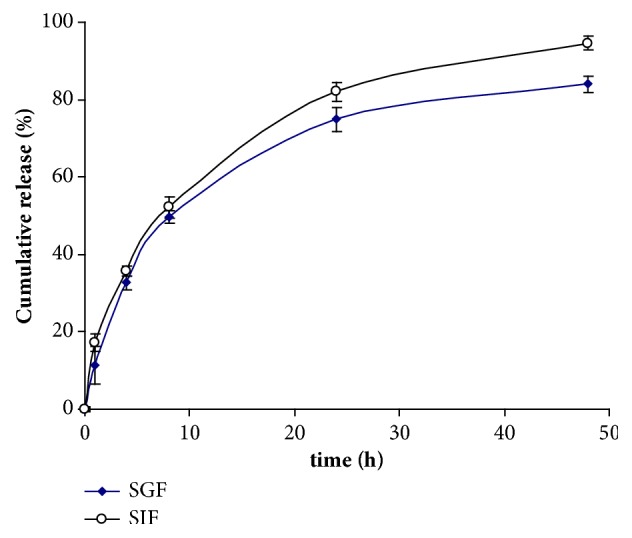
*In vitro* release of plumbagin from oleic acid-based nanoemulsion (polysorbate 80 (3.5%) as a function of time. SGF, simulated gastric fluid (SGF), and simulated intestinal fluid (SIF) at 37°C was used as release media.

The plumbagin-loaded formulation (Z-average, 135 nm) was further studied for* in vitro* release in media mimicking physiological conditions: simulated gastric fluid (SGF) and simulated intestinal fluid (SIF). The plumbagin-loaded nanoemulsion showed a one phase exponential release profile of plumbagin with a half-life of 6.1 h in simulated gastric fluid (SGF) and 7.0 h in simulated intestinal fluid (SIF) (Figure 5). Release trends showed a slightly higher span and plateau for SIF compared to SGF, corresponding to the higher ionization of the phenolic hydroxyl group of plumbagin leading to facilitated release in media with higher pH.

### 3.4. In Vitro Cytotoxicity of Plumbagin-Loaded Nanoemulsion

**Figure 6:**
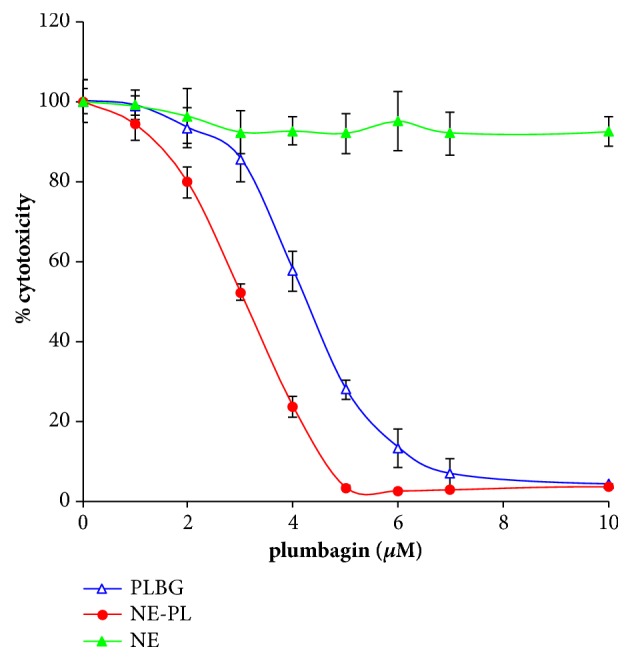
Plumbagin-loaded nanoemulsion shows increased antiproliferative activity. Cytotoxicity of free plumbagin (PLBG), plumbagin-loaded nanoemulsions (NE-PL), and control empty nanoemulsions (NE) toward PTEN-P2 cells. For control NE, the cells were exposed to the same concentration of nanoemulsion. PTEN-P2 cells were incubated with increasing dilutions of formulations for 24 h and then cytotoxicity was determined by cell proliferation assay (MTS).

**Figure 7:**
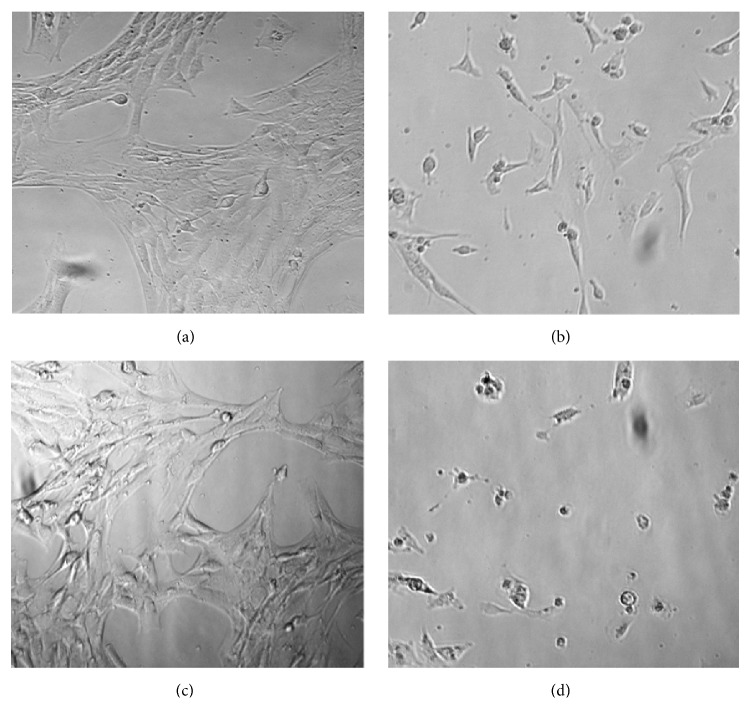
Bright field microscopy images of PTEN-P2 cells. Images show untreated control (a), and cells exposed for 24 h to free plumbagin 4 *μ*M (b), empty nanoemulsion (c), and plumbagin-loaded (4 *μ*M) nanoemulsion (d).

The antiproliferative effect of the plumbagin-loaded nanoemulsion formulation was evaluated using prostate cancer PTEN-P2 cells* in vitro*. Plumbagin-loaded nanoemulsion and free plumbagin inhibited PTEN-P2 proliferation in dose-dependent manner. However, the plumbagin-loaded nanoemulsion showed increased cytotoxicity toward PTEN-P2 cells compared to free plumbagin, IC_50_(*μ*M) 3.1 versus 4.3 after 24 hr exposure (Figure 6). These observations correspond to microscopic assessment, where plumbagin-loaded nanoemulsion apparently caused more cellular damage to PTEN-P2 cells compared to free plumbagin (Figure 7). Besides a decrease in cell density, morphological changes included cell detachment and shrinkage (Figure 7). Control nanoemulsions without plumbagin did not show significant cytotoxicity (Figures 6 and 7).

## 4. Discussion

It is well recognized that the aqueous solubility of a drug is a fundamental property that controls the rate and extent of its absorption and bioavailability [17]. Therefore, as a limiting factor of bioavailability, it essentially influences the pharmacokinetic profile and the pharmacodynamics of the drug after oral administration. Since the majority of the drug candidates in drug discovery show poor aqueous solubility [22], this is a significant challenge. To address this issue, several approaches have been developed, including formulation of poorly soluble drugs in nanoparticle-based drug delivery systems (polymeric micelles, liposomes, nanoemulsions, solid lipid nanoparticles, etc.). Among these, nanoemulsions provide the documented advantage of increased absorption and bioavailability, enhanced penetration via biological membranes, and lower inter- and intraindividual variability in drug pharmacokinetics [19, 20].

We have generated plumbagin-loaded oleic-acid based nanoemulsions using high pressure homogenization of oleic acid emulsion with polysorbate 80 as emulsifying agent. Polysorbate 80, a nonionic surfactant with a high hydrophilic-lipophilic balance (HLB=15.0), was chosen to ensure immediate formation of oil-in-water droplets during production. Depending on surfactant content (1.0-3.5% (w/w)), the high pressure homogenization process yielded plumbagin-loaded nanoemulsions with stable size distribution profiles in the range of 135-220 nm. High pressure homogenization methodology is implemented on a large scale in the pharmaceutical industry and established procedures will provide a distinctive advantage when production of higher quantities of nanoemulsions is needed.

The plumbagin-loaded nanoemulsion (135 nm Z-average, 10% (w/w) oleic acid, 3.5% (w/w) polysorbate 80) which had a good retention of size distribution over time and polydispersity was selected for further studies of drug release, stability in simulated physiological fluids, and* in vitro* cytotoxicity against prostate cancer cells. The plumbagin-loaded nanoemulsion showed good retention of nanoparticle size after dispersion in water or media simulating physiological environment. Furthermore, drug release studies showed exponential release of plumbagin from nanoemulsion with half-lives that would permit optimal absorption of plumbagin from the gastrointestinal tract. Plumbagin-loaded nanoemulsion exhibited higher antiproliferative activity on PTEN-P2 cells compared to free plumbagin. Of note, there is no reason to think that plumbagin released from the nanoemulsion would act differently in prostate cancer cells compared to other types of cancer cells; therefore it is expected that once released, plumbagin will have the same effects as have been extensively described in the literature. Increased cytotoxicity of plumbagin-loaded nanoemulsion was not due to an additive effect, because the control nanoemulsion (without plumbagin) did not show apparent cytotoxicity at the equivalent dose of components. Therefore, the increased cytotoxicity of plumbagin in the nanoemulsion formulation could be attributed either to increased cellular uptake of plumbagin in the nanoparticulate form or to a stabilizing effect of nanoemulsions on plumbagin or both. The high particle surface area [20] associated with small diameter of nanodispersion (surface to mass ratio) could lead to increased reactivity with cellular surface and thus increased uptake of plumbagin by PTEN-P2 cells. Moreover, plumbagin as an electrophilic compound has a propensity to react with various nucleophiles in the extracellular environment. The shielding of plumbagin in the hydrophobic core of the nanoemulsion would therefore decrease its exposure and potential interaction with nucleophiles before reaching cellular targets. This stabilizing effect would be expected to improve the pharmacokinetic/pharmacodynamic behavior of plumbagin when administered using a nanoemulsion formulation.

The nanoemulsion described here was formulated using oil and surfactant components that are biocompatible and generally recognized as safe (GRAS). Additionally, the oleic acid is an FDA-approved excipient and penetration enhancer. High loading capacity for active pharmaceutical ingredient of interest (plumbagin), retention of nanoparticle size over time, kinetics of plumbagin release under simulated physiological conditions, and antiproliferative activity all indicate that the oleic acid-based nanoemulsion formulation is a promising drug delivery system for plumbagin.

## Figures and Tables

**Table 1:** Changes in particle size distribution after dispersion of nanoemulsions in media simulating physiological conditions. Control, empty (NE), and plumbagin-loaded (NE-PL) nanoemulsions (3.5% polysorbate 80) were dispersed in indicated media (1000 x dilution) and incubated for 24 h at 37°C. The particle size distribution was determined by DLS. PDI values are indicated in brackets.

	Water	HCl 0.1 M	0.01 M NaH_2_PO_4_/Na_2_HPO_4_	
			pH 6.8	pH 7.5
NE, empty	134.5 (0.111)	134.2 (0.115)	126.6 (0.127)	158.8 (0.177)
NE-PL, loaded	135.1 (0.166)	135.2 (0.101)	135.7 (0.088)	185.7 (0.149)

## Data Availability

All data are available at Vaccine Research Institute of San Diego, San Diego Science Center, San Diego, CA.

## Abbreviations

DLS: Dynamic light scattering
HLB: Hydrophilic-lipophilic balance
S: Surfactant
NE: Nanoemulsions
NE-PL: Plumbagin-loaded nanoemulsions
PDI: Polydispersity index
PLBG: Plumbagin
SGF: Simulated gastric fluid
SIF: Simulated intestinal fluid.

## References

[1] Padhye S., Dandawate P., Yusufi M., Ahmad A., Sarkar F. H. (2012). Perspectives on medicinal properties of plumbagin and its analogs. *Medicinal Research Reviews*.

[2] Liu Y., Cai Y., He C., Chen M., Li H. (2017). Anticancer Properties and Pharmaceutical Applications of Plumbagin: A Review. *American Journal of Chinese Medicine*.

[3] Panichayupakaranant P., Ahmad M. I. (2016). Plumbagin and Its Role in Chronic Diseases. *Drug Discovery from Mother Nature*.

[4] Yan W., Wang T.-Y., Fan Q.-M. (2014). Plumbagin attenuates cancer cell growth and osteoclast formation in the bone microenvironment of mice. *Acta Pharmacologica Sinica*.

[5] Raja Naresh R. A., Udupa N., Uma Devi P. (1996). Niosomal Plumbagin with Reduced Toxicity and Improved Anticancer Activity in BALB/C Mice. *Journal of Pharmacy and Pharmacology*.

[6] Cao Y., Yu J., Liu T. (2018). Plumbagin inhibits the proliferation and survival of esophageal cancer cells by blocking STAT3-PLK1-AKT signaling. *Cell Death & Disease*.

[7] Kang C. G., Im E., Lee H.-J., Lee E.-O. (2017). Plumbagin reduces osteopontin-induced invasion through inhibiting the Rho-associated kinase signaling pathway in A549 cells and suppresses osteopontin-induced lung metastasis in BalB/c mice. *Bioorganic & Medicinal Chemistry Letters*.

[8] Sunil Kumar M. R., Kiran Aithal B., Udupa N. (2011). Formulation of plumbagin loaded long circulating pegylated liposomes: In vivo evaluation in C57BL/6J mice bearing B16F1 melanoma. *Drug Delivery*.

[9] Sinha S., Pal K., Elkhanany A. (2013). Plumbagin inhibits tumorigenesis and angiogenesis of ovarian cancer cells in vivo. *International Journal of Cancer*.

[10] Xu K.-H., Lu D.-P. (2010). Plumbagin induces ROS-mediated apoptosis in human promyelocytic leukemia cells *in vivo*. *Leukemia Research*.

[11] Abedinpour P., Baron V. T., Chrastina A. (2017). Plumbagin improves the efficacy of androgen deprivation therapy in prostate cancer: A pre-clinical study. *The Prostate*.

[12] Abedinpour P., Baron V. T., Chrastina A., Welsh J., Borgström P. (2013). The combination of plumbagin with androgen withdrawal causes profound regression of prostate tumors in vivo. *The Prostate*.

[13] Rondeau G., Abedinpour P., Chrastina A., Pelayo J., Borgstrom P., Welsh J. (2018). Differential gene expression induced by anti-cancer agent plumbagin is mediated by androgen receptor in prostate cancer cells. *Scientific Reports*.

[14] Hafeez B. B., Zhong W., Fischer J. W. (2013). Plumbagin, a medicinal plant (Plumbago zeylanica)-derived 1,4-naphthoquinone, inhibits growth and metastasis of human prostate cancer PC-3M-luciferase cells in an orthotopic xenograft mouse model. *Molecular Oncology*.

[15] Hafeez B. B., Zhong W., Mustafa A., Fischer J. W., Witkowsky O., Verma A. K. (2012). Plumbagin inhibits prostate cancer development in TRAMP mice via targeting PKC*ε*, Stat3 and neuroendocrine markers. *Carcinogenesis*.

[16] Hafeez B. B., Fischer J. W., Singh A. (2015). Plumbagin inhibits prostate carcinogenesis in intact and castrated pten knockout mice via targeting PKC , Stat3, and epithelial-to-mesenchymal transition markers. *Cancer Prevention Research*.

[17] Amidon G. L., Lennernäs H., Shah V. P., Crison J. R. (1995). A Theoretical Basis for a Biopharmaceutic Drug Classification: The Correlation of in Vitro Drug Product Dissolution and in Vivo Bioavailability. *Pharmaceutical Research*.

[18] Gupta A., Eral H. B., Hatton T. A., Doyle P. S. (2016). Nanoemulsions: formation, properties and applications. *Soft Matter*.

[19] Wang X., Jiang Y., Wang Y.-W., Huang M.-T., Ho C.-T., Huang Q. (2008). Enhancing anti-inflammation activity of curcumin through O/W nanoemulsions. *Food Chemistry*.

[20] Salvia-Trujillo L., Soliva-Fortuny R., Rojas-Graü M. A., McClements D. J., Martín-Belloso O. (2017). Edible Nanoemulsions as Carriers of Active Ingredients: A Review. *Annual Review of Food Science and Technology*.

[21] Jiao J., Wang S., Qiao R. (2007). Murine cell lines derived from Pten null prostate cancer show the critical role of PTEN in hormone refractory prostate cancer development. *Cancer Research*.

[22] Kawabata Y., Wada K., Nakatani M., Yamada S., Onoue S. (2011). Formulation design for poorly water-soluble drugs based on biopharmaceutics classification system: basic approaches and practical applications. *International Journal of Pharmaceutics*.

